# Knowledge differences on the impact of age on fertility among fertile and subfertile women

**DOI:** 10.5935/1518-0557.20240112

**Published:** 2025

**Authors:** Waranya Rugfoong, Jamjit Doungpunta, Natpat Jansaka, Usanee Sanmee

**Affiliations:** 1 Division of Reproductive Medicine, Department of Obstetrics and Gynecology, Faculty of Medicine, Chiang Mai University, Chiang Mai 50200, Thailand; 2 CMEx Fertility Center, Center of Medical Excellence, Chiang Mai University, Chiang Mai 50200, Thailand

**Keywords:** age and fertility, knowledge, fertile, subfertile, women

## Abstract

**Objective:**

To explore the knowledge concerning the impact of age on fertility in Thai women among fertile and subfertile women.

**Methods:**

A cross-sectional study was conducted at Maharaj Nakorn Chiang Mai Hospital, Thailand. The questionnaires consisted of sociodemographic questions and knowledge pertinent to the impact of age on fertility. Both fertile and subfertile women completed the same questionnaire anonymously.

**Results:**

A total of 500 women were included in the study, with two hundred and fifty in each group. The majority of women in both the fertile and subfertile group correctly identified the most fertile age of women (84.8% *vs* . 84.4%, *p* =0.902) and the impact of age on infertility treatment (73.2% *vs* . 84.0%, *p* =0.003). However, only one-fifth of the participants in both groups correctly identified the age that their fertility starts to decline, show a marked decline and ends. Most women in both groups chose the answer that overestimates their fertility potential. Moreover, 10.8% of subfertile women believed that fertility is not dependent on age and 16.0% believed that age does not affect the success rate of infertility treatment.

**Conclusions:**

The overall knowledge of women regarding the impact of age on fertility is relatively poor with no difference between fertile and subfertile women. These findings may indicate an urgent need to increase this area of knowledge in the population.

## INTRODUCTION

Age is the most important factor in a woman’s fertility. Women have an optimal reproductive window and a reproductive endpoint that is determined by nature. Women experience an increased incidence of infertility as they age, from 6% at the age of 20-24 years to 64% at the age of 40-44 years ( Menken *et al.* , 1986 ). Moreover, age is considered the best predictor in the success of infertility treatment. It has been found that the live birth rate per embryo transfer was 40% in women under 30 years of age dropping to only 11% in women aged 41-42 years ( ACOG, 2014 ). Therefore, it can be concluded that a woman’s age is a very important factor in having children both naturally and through assisted reproductive technology (ART).

Nowadays, women are choosing to delay having children until later ages when compared with previous generations. Knowledge about the impact of age on fertility is therefore important and necessary for all woman to plan for future childbearing.

Studies have found that women’s knowledge about age affecting fertility is poor. Most women still believe that even at their advanced age they can easily have children and the expectation of success from infertility treatment is higher than real outcome data. They believe that ART makes it possible to have children even at an advanced age. Interestingly, the results of the study were in the same direction as have been discovered in the general population ( Deatsman *et al.* , 2016 ; Gossett *et al.* , 2013 ; Hashiloni-Dolev *et al.* , 2011 ; Sarría-Santamera *et al.* , 2020 ), and in medical personnel including obstetrician-gynecologists ( Fritz *et al.* , 2018 ; García *et al.* , 2017 ; Yu *et al.* , 2016 ).

We were interested in studying the knowledge regarding the impact of age on fertility in Thai women and wanted to question that whether the ability to conceive was influenced by this knowledge by comparing fertile and subfertile women.

## MATERIALS AND METHODS

This cross-sectional study was carried out in Maharaj Nakorn Chiang Mai Hospital, Chiang Mai, Thailand from August 2023 to May 2024 with the approval of the Research Ethics Committee of the Faculty of Medicine, Chiang Mai University.

We recruited 500 females aged between 18 and 50 years, who were either pregnant for the first time or primary infertile with sufficient Thai language proficiency to complete a questionnaire-based survey. Women were approached in two settings, the CMEx Fertility Center and Hospital Antenatal care unit. Those who voluntarily filled in the questionnaire were considered as giving their consent. Both pregnant and subfertile women completed the same questionnaire anonymously.

The questionnaire comprised two parts. The first part was designed to record the sociodemographic background including age, years lived with partner, years trying to conceive, education, occupation, income per month and religion. The second part assessed their knowledge on the impact of age on fertility. Single best answer questions were asked about the age at which women are most fertile, the age when fertility starts to decrease, the age when fertility markedly decreases, the age when fertility ends and the impact of age on the success of in vitro fertilization (IVF). Only completely filled-out questionnaires were used for data analysis.

The data are presented as number and percentage, mean and standard deviation, or median and interquartile range. Data was analyzed using the Chi-square test, independent sample t-test or Mann-Whitney U test based on data type and distribution using the SPSS program version 27. A *p* -value<0.05 was considered statistically significant.

## RESULTS

A total of 500 women were included in the study separated into two hundred and fifty women in the pregnant (fertile) and the subfertile group. There was a significant difference in the sociodemographic background regarding age, years lived with partner, years trying to conceive, education, occupation and income per month between fertile and subfertile women ( Table 1 ). Women in the subfertile group were on average 7.3 years older, had lived with their partner 2.6 years longer and had been trying to conceive 0.7 years longer than those in the fertile group ( *p* <0.001). They also have a higher level of education and more income per month than those in the fertile group ( *p* <0.001). Most of the subfertile women (33.6%) had their own business, while most fertile women (40.0%) were private officer. There were no differences in religion between these two groups. The majority of the participants were Buddhist ( Table 1 ).

**Table 1:** Differences in participant demographics of fertile and subfertile women.

Parameters	Fertile (n=250)	Subfertile (n=250)	p-value
Age (years) ^*^	28.5±4.8	35.8±5.2	< 0.001
Years lived with partner ^†^	2.4 (1.1 - 6.0)	5.0 (3.0 - 10.0)	< 0.001
Years tried to conceive ^†^	2.3 (2.2 - 2.6)	3.0 (2.7 - 3.3)	< 0.001
Education Under bachelor’s degree Bachelor’s degree Higher than a bachelor’s degree	82 (32.8) 158 (63.2) 10 (4.0)	63 (25.2) 140 (56.0) 47 (18.8)	0.061 0.101 <0.001
Occupation Government officer Private officer Personal business Others	36 (14.4) 100 (40.0) 48 (19.2) 66 (26.4)	73 (29.2) 45 (18.0) 84 (33.6) 48 (19.2)	<0.001 <0.001 <0.001 0.055
Income per month (baht) ^‡^ ≤ 10,000 10,001-20,000 20,001-30,000 > 30,000	40 (16.0) 141 (56.4) 54 (21.6) 15 (6.0)	21 (8.4) 83 (33.2) 76 (30.4) 70 (28.0)	0.010 < 0.001 0.025 < 0.001
Religion Buddhist Christian Muslim Others	220 (88.0) 25 (10.0) 4 (1.6) 1 (0.4)	225 (90.0) 23 (9.2) 2 (0.8) 0	0.476 0.762 0.413 0.319

Values are frequency, n (%), Chi-square test, unless otherwise stated

* Mean±standard deviation, t-test

† Median (interquartile range), Mann-Whitney U test

‡ US Dollar conversion rate was 36 baht to 1 US Dollar at the time of data collection

Regarding the knowledge on the impact of age on fertility, the majority of women in both the fertile (84.8%) and subfertile group (84.4%) correctly identified that the most fertile age of women is before the age of 30 years ( *p* =0.902, Table 2 ). Only 15.6% of participants correctly recognized that their fertility starts to decrease at the age of 30-34 years (13.2% in the fertile and 18.0 % in the subfertile group, *p* =0.140). The majority of women in both groups (40.8% in the fertile and 48.8% in the subfertile group) believed that their fertility starts to decrease at the age of 35-39 years. Only 18.4% of participants correctly identified that their fertility shows a marked decrease at the age of 35-39 years with more correct answers in the subfertile group (22.4% *vs* . 14.4%, *p* =0.021). The majority of women in both groups (37.6% in the fertile and 40.4% in the subfertile group) believed that their fertility had a marked decrease at the age of 40-44 years. An average of only 23.4% of participants correctly recognized that their fertility ends at the age of 45-49 years (20.4% in the fertile and 26.4% in the subfertile group, *p* =0.114). The majority of women in both groups (57.6% in the fertile and 52.0% in the subfertile group) believed that their fertility ends after the age of 50 years. Around 11.0% of participants believed that fertility is not dependent on age (11.2% in the fertile and 10.8% in the subfertile group). Most women in both groups correctly identified that the age of a women affects the rate of IVF success with more correctly answering in the subfertile group (73.2% *vs* . 84.0%, *p* =0.003). However, 21.4% of participants (26.8% in the fertile and 16.0% in the subfertile group) believed that IVF success rate is not affected by age ( Table 2 ).

**Table 2:** Knowledge regarding the impact of age on fertility in fertile and subfertile women.

Questions	Fertile (n=250)	Subfertile (n=250)	p-value^*^
At what age are women the most fertile? Before 30 ^†^ 30 - 34 35 - 39 40 - 44 45 - 49 > 50 No effect	212 (84.8) 27 (10.8) 6 (2.4) 2 (0.8) 1 (0.4) 0 2 (0.8)	211 (84.4) 26 (10.4) 9 (3.6) 2 (0.8) 0 0 2 (0.8)	0.902
At what age does female fertility start to decrease? Before 30 30 - 34 ^†^ 35 - 39 40 - 44 45 - 49 > 50 No effect	4 (1.6) 33 (13.2) 102 (40.8) 62 (24.8) 30 (12.0) 14 (5.6) 5 (2.0)	3 (1.2) 45 (18.0) 122 (48.8) 59 (23.6) 14 (5.6) 4 (1.6) 3 (1.2)	0.140
At what age is there a marked decrease in female fertility? Before 30 30 - 34 35 - 39 ^†^ 40 - 44 45 - 49 > 50 No effect	3 (1.2) 8 (3.2) 36 (14.4) 94 (37.6) 51 (20.4) 50 (20.0) 8 (3.2)	0 13 (5.2) 56 (22.4) 101 (40.4) 53 (21.2) 26 (10.4) 1 (0.4)	0.021
At what age does fertility end in women? Before 40 40 - 44 45 - 49 ^†^ > 50 No effect	5 (2.0) 22 (8.8) 51 (20.4) 144 (57.6) 28 (11.2)	2 (0.8) 25 (10.0) 66 (26.4) 130 (52.0) 27 (10.8)	0.114
Does the age of women affect the success rate of *in-vitro* fertilization (IVF)? Yes ^†^ No	183 (73.2) 67 (26.8)	210 (84.0) 40 (16.0)	0.003

Values are frequency, n (%)

* Chi-square test, compare the proportion of fertile and subfertile women who responded with the correct answer

† Correct answer

## DISCUSSION

Nature determines that women have a maximum number of oocytes of 6-7 million at 20 weeks of gestation with no further increase in number during development. This number will gradually decrease to 1-2 million oocytes at birth, to 300,000-500,000 oocytes when entering puberty, 25,000 oocytes at age 37 years and remain at approximately 1,000 oocytes during menopause ( Faddy *et al.* , 1992 ). In addition to the number of oocytes decreasing with age, oocyte quality also deteriorates with age. It has been found that embryos created from ART have more chromosomal abnormalities as women age, specifically a 23%, 35% and 58% aneuploidy rate in 30-, 35- and 40-year-old women respectively, and as high as 84% in 45-year-olds ( Franasiak *et al.* , 2014 ). Both oocyte number and quality show strong correlation to age, causing women’s fertility to begin to decline after age 30 years with a more rapid decline after age 35 years ( Utting & Bewley, 2011 ). Fertility is considered to end at an average age of 45 years ( Leridon, 2004 ) before menopause begins at around age of 50 years. We used the previous research in this natural fertility background to evaluate the knowledge of the participants. If women do not realize this fact, it may lead to unintended childlessness in the future. This is especially important in the light of this current trend that women choose to delay childbearing until late 30’s to early 40’s years of age which is the age that their ability to conceive downtrends. This is the reasoning behind why the American College of Obstetricians and Gynecologists and the American Society for Reproductive Medicine makes the recommendations for women to receive fertility evaluation after 6 months of failed attempts to conceive in women older than 35 years or undergo earlier evaluation in women older than 40 years ( ACOG, 2014 ).

A systematic review ( García *et al.* , 2018 ) of this subject included 41 studies on the knowledge of age-related fertility decline in women and found that the majority of the population, whether male or female, fertile or subfertile, non-medical or even medical personnel have a low level of this knowledge. Many studies show that delayed childbearing is the result of a wrong perception about fertility, resulting in a lack of awareness of planning for childbearing ( Cooke *et al.* , 2012 ; Sarría-Santamera *et al.* , 2020 ) which is more likely to result in infertility, increase demand for ART treatment and may end up in an unwanted situation without children. A study by Utepova *et al.* (2019) shows that 80.5% of women over 40 years of age stated that if they had proper knowledge about the effect of age on fertility, they would have tried to have children earlier and not wait until they were this old. We question whether the status of the ability to conceive was influenced by this age-related fertility knowledge. Therefore, we examined the difference in the knowledge between fertile and subfertile women, only including women attempting to have a first child, undergoing a first-time natural pregnancy or primary infertile women. We found that fertile and subfertile women generally made similar answers. Eighty four percent of women in both groups knew that the most fertile age of women is before the age of 30 years. However, most women in both groups estimated the age fertility starts to decline, shows a marked decline and the age fertility ends too late. Their answers were at 35-39 years, 40-44 years, and > 50 years, when the actual ages are 30-34 years, 35-39 years, and 45-49 years. The women appearing to overestimate their reproductive ability. Only one-fifth of the women in both groups correctly answered the age at which fertility declines and ends. This number is consistent with previous studies from Europe ( Chelli *et al.* , 2015 ; Nouri *et al.* , 2014 ; Rovei *et al.* , 2010 ; Virtala *et al.* , 2011 ) and America ( Sabarre *et al.* , 2013 ; Yu *et al.* , 2016 ). There is no relationship between the current conception status and the level of knowledge. Fertile women do not have a higher level of knowledge on age-related fertility decline. Whereas women in the subfertile group have more correctly answered some questions such as the age that women show a marked decrease in fertility and the impact of age on ART treatment.

Although knowledge is no different between fertile and subfertile women, there is a significant difference in their sociodemographic background. It is not surprising that women in the subfertile group were much older than fertile women. The mean age in the subfertile group is within a period of the marked decline of fertility (35.8±5.2 years) while in the fertile group is in the most fertile age for fertility (28.5±4.8 years). Our data support the previous study that reported that women with higher education and occupational status were more likely to be diagnosed with infertility ( Datta *et al.* , 2016 ). This may be attributed to the current social, cultural and economic changes encouraging modern women to have a higher level of education and earn a good income before having a child.

The big concern from our finding is that 11.0% of women, whether fertile or subfertile, had the perception that age does not affect their natural fertility. Moreover, 26.8% of fertile and 16.0% of subfertile women thought that the IVF success rate is not affected by age. They have a false belief that ART treatment can fully compensate for their unsuccessful natural fertility. These misperceptions can lead to childlessness in subfertile women or not having another child in a fertile one. Therefore, it is of great importance to increase the knowledge regarding the impact of age on fertility in the population in particular in young women who wish to become parents in future. This approach is considered as one of the primary prevention strategies for untreatable infertility in the future from age-related fertility decline. The most effective way to fill the gaps in knowledge about the impact of age on fertility is to combine all available instruments both directly through school education beginning in adolescence, and through primary health care providers who are frequently at the front line of health care for patients and also indirectly through mass media, social media and social campaign. Women need accurate knowledge regarding fertility to allow them to make informed reproductive life plans. Although choosing to delay childbearing is a personal preference, it must be a decision made with full knowledge regarding age and their fertility consequences.

In conclusion, this study demonstrated that the overall knowledge of women regarding the impact of age on fertility is relatively poor with no difference between fertile and subfertile women. These may indicate an urgent need to increase this area of knowledge in the population aiming to reduce the prevalence of infertility and unintentional childlessness in new generations.
